# The complete chloroplast genome sequence of *Keteleeria hainanensis* (Pinaceae)

**DOI:** 10.1080/23802359.2019.1662749

**Published:** 2019-09-06

**Authors:** Dong-Lin Li, Yong Yang, Shan Yang, Yu-Kai Chen

**Affiliations:** a College of Ying-Tong Agricultural Science and Engineering, Shaoguan University, Shaoguan, Guangdong, China;; b Ministry of Education, Key Laboratory for Ecology of Tropical Islands, College of Life Sciences, Hainan Normal University, Haikou, China

**Keywords:** *Keteleeria hainanensis*, endemic, chloroplast genome, phylogenomic tree

## Abstract

*Keteleeria hainanensis* is an endemic species with extremely narrow distribution. In this study, the complete genome of *K._hainanensis* was sequenced and analyzed. The genome size is 117,366 bp and it contains two short inverted repeat regions of 1,272 bp, which was separated by a large single-copy (LSC) region of 74,819 bp and a small single-copy (SSC) region of 40,003 bp. The GC content of this genome was 38.57%. The chloroplast genome contained 103 unique genes, including 74 protein-coding gene, 25 tRNA genes, and 4 rRNA gene. Phylogenetic analysis base on 11 chloroplast genomes indicated that *K. hainanensis* is closely related to *K. davidiana.*

The genus Keteleeria in the family Pinaceae is endemic in Eastern Asia, including 12 species and 2 variants in the world. There are 10 species and 2 variants in China, which is the endemic and diverse center (Wang et al. 2012; Lin et al. 2014). Keteleeria species are famous and unique timber species and ornamental plant species. Most Keteleeria species are listed as rare, gradually Endangered or Endangered species by IUCN, CSG and CPRDB (Fu 1992). *Keteleeria hainanensis* Chun et Tsiang, an endemic species, with extremely narrow distribution, occurs only at high altitudes (1100 ∼ 1400 m) in Bawangling on Hainan island (Chen et al. 2011). *Keteleeria hainanensis* is a valuable and excellent tropical timber species for buildings, bridges, furniture, cabins, panels, farm tools, etc. because it has many characteristics such as straight trunk, straight wood texture, heavy material, and strong corrosion-resistance (Mou et al. 2012). Unfortunately, due to long term use and unreasonable harvesting, natural resources have been depleted. At present, there are a few researches on *K. hainanensis*, only a few papers related to its communities (Chen et al. 2011) and chemical composition (Song et al. 2015). In this study, we reported and characterized the complete chloroplast genome sequence of *K. hainanensis* to contribute to further phylogenetical and protective studies of this plant.

The fresh leaves of *K. hainanensis* were collected from Bawangling national nature reserve, Hainan Island (N19°05′48, E109°10′) in China and voucher herbarium specimens were deposited at the Herbarium of Shaoguan University with the accession number Li-201903. Total DNA was extracted from the fresh leaves using the modified CTAB method (Doyle 1987) and then it was sequenced using HiSeq4000 platform of Illumina. In total, 10.5 G raw reads were obtained. The chloroplast genome was assembled using the program NOVOPlasty 3.1 (Dierckxsens et al. 2017) with a part of rbcL gene sequence of *K. davidiana* (NC011930). The assembled chloroplast genome sequence was then annotated using DOGMA (Wyman et al. 2004), coupled with manual check and adjustment.

The complete chloroplast genome of *K. hainanensis* (GenBAnk Accession No. MN180080) was 117,366 bp in length. The genome was a typical quadripartite structure and contained two short inverted repeat regions of 1272 bp, which was separated by a large single-copy (LSC) region of 74,819 bp and a small single-copy (SSC) region of 40,003 bp. The GC content of this genome was 38.57%. There were 103 predicted gene, including 74 protein-coding genes, 25 tRNA genes, and 4 rRNA genes.

A phylogenetic analysis was conducted to confirm the relationship of *K. hainanensis*, 11 complete chloroplast genome sequence of Pinaceae were aligned using MEGA7. A maximum likelihood tree (ML) was constructed using GTR + G+I model with 1000 bootstrap replicates with *Cathaya argyrophylla* as an outgroup (Figure 1). The result showed that *K. hainanensis* is closely related to *Keteleeria davidiana.* Our result will provide valuable information for genetic evolution and molecular study of this Endangered plant.

**Figure 1.**
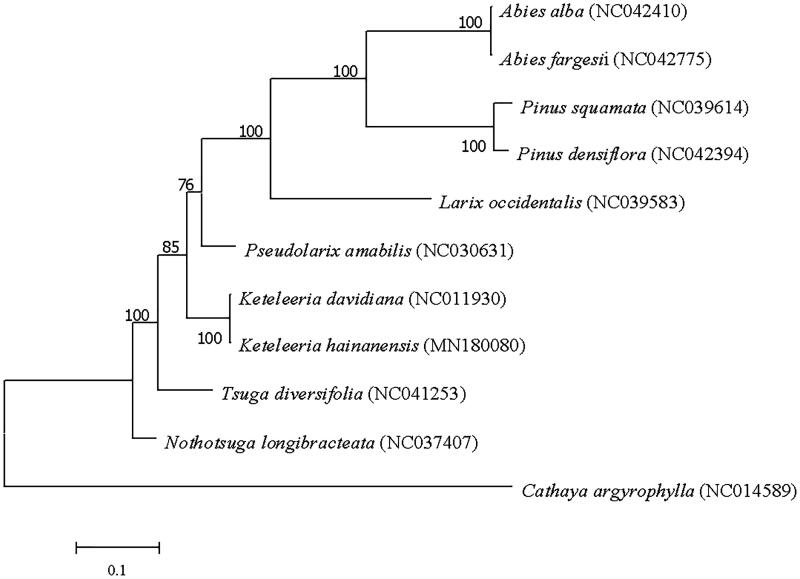
Maximum likelihood tree based on the sequences of eleven complete chloroplast genomes. Numbers in the nodes were bootstrap values from 1000 replicates. Scale in substitutions per site.
